# 100 Steps of a DIEP Flap—A Prospective Comparative Cohort Series Demonstrating the Successful Implementation of Process Mapping in Microsurgery

**DOI:** 10.1097/GOX.0000000000002016

**Published:** 2019-01-15

**Authors:** Hrsikesa R. Sharma, Warren M. Rozen, Bhagwat Mathur, Venkat Ramakrishnan

**Affiliations:** From the * St Andrew’s Centre for Plastic Surgery and Burns, Broomfield Hospital, Mid Essex Hospital Services NHS Trust, Chelmsford, Essex, United Kingdom; † Anglia Ruskin University Chelmsford Campus, Bishops Hall Lane, Chelmsford, United Kingdom.

## Abstract

**Background:**

The demand to improve the efficiency of microsurgical breast reconstruction is driven by increasing number of breast cancer and risk reducing cases, and the concurrent requirement for hospitals to cut costs. Businesses have successfully used process mapping as a tool to improve efficiency; however, process mapping has been sparsely used in surgery. This prospective cohort study has used process mapping to break down the individual components of a deep inferior epigastric artery perforator (DIEP) flap operation into a template of 100 streamlined steps.

**Methods:**

Through observation of the senior author’s uniform technique, refined from experience of over 5,000 cases, the DIEP flap operation was broken down into 100 individual steps, all arranged in a logical sequence with which to maximize efficiency and outcome. This created a 100-step process-mapped template. Subsequently, 2 cohorts of 10 unilateral DIEP cases were prospectively timed. One cohort following this process mapped template and the other control group was blinded to the template.

**Results:**

The process-mapped cohort was 56.1 minutes quicker than the control cohort, despite the addition of symmetrizing surgery being performed concurrently in 4 out of the 10 cases. Furthermore, there was no return to theater in the process-mapped cohort versus 1 return to theater in the control cohort with no flap loss in either group.

**Conclusions:**

This study uniquely presents an approach to process map the DIEP flap operation and demonstrates its utility in improving operative efficiency, without compromising outcomes. It also illustrates the possibility of symmetrizing surgery being carried out through parallel operating processes, without affecting overall operative times.

## INTRODUCTION

Process mapping is an important technique used in a range of industries to facilitate work flow, and this is exemplified in health care, where an aging and increasing population meets increased cost demands of this changing demographic. Process mapping involves breaking down a process into smaller steps, each of which is further subdivided until all the individual components have been mapped. By carrying out each of the steps in an efficient and logical manner, outcomes may be assessed and improved. The leading Formula One motor sport teams, for example, routinely and repeatedly are able to change the 4 tyres of their cars during pit stops in 3 seconds. This involves a team of people each having individual roles that have been optimized, leading to a more efficient overall maneuver.

We postulate that the same principles of process mapping can be successfully applied to surgical procedures, aiming to maximize efficiency and thus save time and costs. This will be beneficial for patients as they have shorter anesthetic times and may even have multiple procedures done during the same operation as opposed to 2 separate operations. Furthermore, shorter operations may result in shorter waiting lists. Indeed, there has been a small amount of data in the literature indicating the beneficial role of process mapping in surgery.^1–3^ However, most of these have been associated with turnover between cases and minimizing interoperative time, that is, time delays between different patients’ operations in the same theater. Fong et al.^4^ demonstrated that there is a paucity of evidence about intraoperative efficiency and process mapping but concluded work in this area this would help improve operative efficiency.

Not explored previously, we aim in the current study to apply a process mapping technique to the process of a deep inferior epigastric artery perforator (DIEP) flap^5^ operation for breast reconstruction. The DIEP flap is widely considered the gold standard procedure in this setting, despite a lengthy operation, and variability in operator ease with the procedure. This was thus a suitable procedure for which to apply process mapping. It has been shown that 2 DIEP operations^6^ can be carried within daytime hours, and more recently our unit has shown that 3 can be carried out in a 12-hour working day^7^ routinely. The use of process mapping seeks to identify the facets that may enable such efficiency to become more mainstream.

## PATIENTS AND METHODS

A prospective cohort study was undertaken using a single institution’s analysis of process mapping in a cohort of patients undergoing DIEP flap breast reconstruction. The aims of this study comprised first to demonstrate the individual steps of a DIEP flap operation, and second to demonstrate that by process mapping the DIEP flap, the efficiency of the operation is improved while not compromising on outcome.

### The Study Design Was As Follows

Initially, the DIEP flap operation had to be process mapped into its individual steps which numbered 100. This was done by using the senior author’s technique (a refinement to a uniform technique based on a total experience of over 5,000 cases of autologous breast reconstruction) as the basis to break down the DIEP flap operation into its individual steps.

This breakdown or process mapping of steps was achieved through observation of several consecutive unilateral DIEP flap operations of the senior author. Each required individual step was recorded from the moment the patient entered the anesthetic room to the moment they were woken up at the end of the procedure. Collating these individual steps in sequence enabled us to define a continuous stream of steps that would encompass the process mapping of the DIEP flap based upon the senior author’s refined technique. A template of the process mapped 100 steps of the DIEP flap is thus presented (see Results).

This template was then used to time 10 consecutive DIEP flap operations of a single surgeon, the senior author. This cohort is based upon the process mapping template. A second cohort of 10 consecutive DIEP flap operations performed by a second senior surgeon, blinded to the process mapping template, was also timed using the template as a control. Results of timings between the 2 cohorts were compared and analyzed to test the aim.

To minimize bias, a single investigator timed each case using the template tool. Only unilateral autologous DIEP reconstructions were included in this study.

### Operative Technique

All included cases were carried out at St Andrew’s Centre for Burns and Plastic Surgery, where over 250 DIEP flap cases are performed annually. Hospital ethical clearance was sought and permission for this study to continue was granted as it was also a review of current practice of 2 senior surgeons and the study was deemed to pose no added danger/risk to the patients as no changes to practice were made and only data documentation was required to carry out this study.

Each case had preoperative computed tomography angiography to select the “best” perforator for the flap. The senior surgeons in both cohorts was the primary operating surgeon in flap raising and anastomosis with 2 senior trainees assisting each case carrying out parallel components such as recipient vessel preparation and abdominal closure. Bilateral, bi-pedicled or stacked cases were excluded from this study. Both immediate and delayed cases were included and timings for mastectomy or excision of mastectomy scar and pocket creation were noted for interest only. The data collected were predominantly nonparametric; hence, statistical analysis was limited.

## RESULTS

### The 100 Steps of a DIEP Flap

#### Anesthetic Room

1. Checklist
2. Lines - venous
3. Laryngeal mask airway (as opposed to endotracheal intervention)—bag and mask and airway control
4. Oesophageal Doppler Insertion (No Arterial line routinely used)
5. Electrocardiography leads attachment and monitoring
6. Catheterization

#### Preparation and Draping

7. Exposure of patient for surgical preparation and draping
8. Positioning—knees and arm securing
9. Flowtron/calf pumps and diathermy pad attachment
10. 2 diathermy machines set up for 2 team operating
11. World Health Organization patient and procedure check
12. Skin preparation
13. Sterile drapes secured
14. Checking of marking/remarking/marking/stapling of midline points

#### Initial Flap Raise

15. Skin incision lower contralateral flap with scalpel
16. Dermal and subcutaneous dissection continued with hand held diathermy
17. SIEV identification
18. SIEV dissected tenotomy forceps and ligaclipping/cauterization depending on caliber/size of vein
19. Dissection down to Scarpa’s fascia with hand held diathermy
20. Subscarpa’s fascia dissection to rectus fascia
21. Skin incision upper contralateral flap
22. Dissection down to Scarpa’s Fascia
23. Sub-Scarpa’s dissection beveled cranially to rectus fascia for fat recruitment/volume recruitment and matching contour for closure
24. Lateral raise of flap off rectus fascia with hand held diathermy to just lateral to lateral row perforator level

#### Perforator Dissection

25. Dissection down to and identification of perforator (matched to computed tomography) using bipolar diathermy and/or McIndoe’s dissecting scissors
26. Umbilical release down to Fascia to aid perforator dissection/superior access to perforator
27. Circling cuff/isolation of perforator above rectus fascia using tenotomy/McIndoe forceps
28. Rectus Fascia incised with Scalpel
29. Subfascial/intramuscular dissection (muscle relaxant versus lignocaine) using McIndoe dissecting forceps and bipolar diathermy (low setting)
30. Submuscular dissection of perforator
31. Identification of DIEA artery
32. Proximal/superior ligation of DIEA artery with ligaclips
33. Distal/inferior pedicle dissection to adequate length (pedicle length noted)
34. Dissection of flap off rectus fascia across midline
35. Pedicle ligated and checking for backflow across midline versus. letting “breath”
36. Ipsilateral lower skin incision ipsilateral dermal and subcutaneous dissection
37. SIEV identification
38. SIEV dissected
39. Dissection down to rectus fascia
40. Superior ipsilateral skin incision
41. Dissection down to Scarpa’s fascia
42. Sub-Scarpa’s dissection and fat recruitment
43. Lateral raise of flap off rectus fascia
44. Flap off
45. Hemostasis of pocket postmastectomy
46. Dissection to identify recipient vessels—internal mammary artery perforator versus thoracodorsal vessels versus internal mammary vessels
47. Macroscopic dissection of recipient vessels
48. Shaping/suturing of pocket
49. Drains
50. Hemostasis

#### Vessel Preparation and Flap Inset

51. Zone 4/3 discarding (Hartrampf and Holm)
52. Hemostasis
53. St Andrew’s Coning suture of under surface of flap using absorbable suture for projection of flap/coning
54. De-epithelialization of flap
55. Hemostasis post de-epithelialization

#### Abdominal Closure

56. Hemostasis subrectus fascia and muscle
57. Mesh (not sutured)
58. Muscle versus no muscle repair
59. Rectus Fascia closure using loop nylon suture
60. Bed break and check for closure tension
61. Abdominoplasty flap raise while checking closure tension (up to xiphisternum)
62. Insertion of one abdominal drain using scalpel incision and drain secured
63. Hemostasis of abdomen
64. Neo-umbilicus marking
65. Skin incision of neo-umbilicus in abdominoplasty flap
66. Cuff of Sub-Scarpa’s fat release around neo-umbilicus from underneath abdominoplasty flap
67. 2.0 Vicryl anchoring sutures between rectus fascia lateral to umbilicus and dermal edge of neo-umbilical incision
68. Scarpa’s fascia closure (3 either side and midline)
69. Dermal closure using barbed suture
70. Umbilical skin closure using 5.0 monocryl subcuticlar/abdomen closed
71. Preneo (Ethicon) tape and glue
72. Abdominal drain opened

#### Microsurgery

73. Self retainers to gain access to recipient vessels (secured with op tape or held by assistant)
74. Flap positioning for micro: stapled/sutured
75. Microscopic venous dissection and clamping
76. Background insertion
77. Venous anastomosis (with or without coupler)
78. Microscopic Arterial Dissection with clamping
79. Arterial anastomosis including removal of clamps and checking for bleeding (micro ligaclips for small leak)
80. Acland’s test to confirm flow of artery and vein
81. Checking of venous bleeding from second DIEV if present before clipping off
82. Assessment of flap bleeding from dermal edge and side of flap
83. Hemostasis of flap

#### Breast/Flap Closure

84. Anchoring sutures for flap 2.0 Vicryl
85. Check pedicle for twisting/kinking
86. Circa skin paddle dermal release
87. Subcuticular closure of flap
88. Subcuticular closure of axillary wound if present/last stitch
89. Preneo Tape and Glue (care not to cover flap skin paddle with glue)
90. Drains opened
91. Check flap for capillary refill

#### Final Transfer

92. Wet and dry clean of wounds
93. Removal of drapes
94. Rolling and transfer
95. Cleaning
96. Binder
97. Arm positioning
98. Check flap posttransfer
99. Pillow support for arm and knees
100. Wake patient up

### Process Mapping Study

The timing results of each 10-case cohort (process mapped versus control) were collected and compared (Table 1), and the outcome of successful flap reconstruction was achieved in all 20 cases.

**Table 1.**
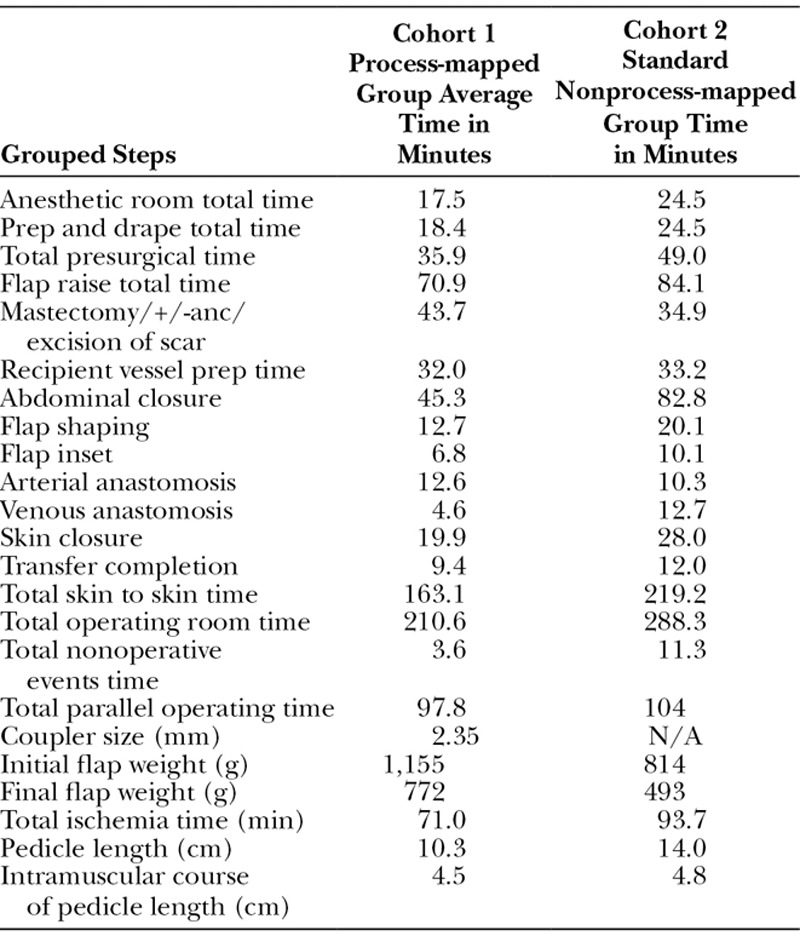
Average Timing Data from 10 Cohort 1 Cases (Process Mapped Group) Versus 10 Cohort 2 Cases (Blinded/Control Group)

#### Demographics

Cohort 1 (Process Mapped) comprised 6 delayed and 4 immediate unilateral DIEP flaps. Of these, 9 were right-sided and 1 left-sided reconstructions. The average age was 46.4 years old (range, 31–58 years) and average BMI 29.25 (range, 24.6–38.5).

Four cases had additional symmetrizing (contralateral breast reduction ×3 and mastopexy ×1) procedures in their timing—3 delayed, and 1 immediate. Of these, 1 delayed case needed an intraoperative vein graft from SIEV to Internal mammary perforator vein as superficial venous system was dominant. There were zero returns to theater and no flap losses.

Cohort 2 (control) comprised 3 delayed and 7 immediate unilateral DIEP flaps. Of these, 8 were right-sided and 2 left-sided reconstructions. The average age was 52 years old (range, 44–67 years), and the average BMI 28.3 (range, 22.9–37.7). No cases had additional symmetrizing procedures. There was one return to theater: in which a delayed case needed a vein graft: cephalic turndown to SIEV. There were no flap losses.

## DISCUSSION

The current study demonstrated that the DIEP flap operation was able to be successfully process mapped into 100 steps, which were able to be consistently applied to the procedure across multiple surgeons. Moreover, the study was able to show that process mapping was useful in identifying areas of variability between surgeons, and areas for assessment within the procedure of a single surgeon.

There were significant differences identified between different groups, when process mapping was applied to DIEP flap surgery as a means to identifying these differences. The aims of the study were thus realized, with specific processes able to be considered in improving efficiency and operative flow and therefore potentially for teaching and for surgical training,

In terms of identifying these differences, cohort 1, which was the process-mapped cohort, had an average skin to skin operative time of 163.1 minutes compared with cohort 2 (control cohort), which averaged 219.2 minutes. Though 4 out of the 10 cases in cohort 1 were immediate DIEP flaps, which may be perceived as taking longer time, these 4 immediate cases in fact averaged only 160 minutes skin to skin time.

The flap raise was quicker in cohort 1 than cohort 2. However, as well as removing redundant steps this could also be partly explained by the fact that the average length of pedicle in cohort 1 was 10.1 cm compared with 14 cm in cohort 2. This is because cohort 1 had anastomoses with internal mammary and internal mammary perforators and thoracodorsal recipients and therefore would not require as long a pedicle on average as if they had solely been anastomosed to the thoracodorsal axis as was the case throughout cohort 2. This is known as the short pedicle raise.

The greatest difference in times between the 2 cohorts was the abdominal closure. Cohort 1 uses a few sutures to close the Scarpa’s fascia and then a barbed suture to close the dermis with tape for the epidermis and on average took 43.7 minutes for closure. This is in comparison to the standard 3-layer closure used in cohort 2, which took 82.8 minutes on average. This demonstrated that with equal results and no wound healing problems of donor sites of 2 groups, the process mapping approach of cohort 1 is the more efficient approach to abdominal closure saving on average 39.1 minutes by streamlining the process of abdominal donor-site closure.

Overall, the process-mapping cohort 1 has saved on average 56.1 minutes per operative time compared with the control cohort 2 group and demonstrate that process mapping the DIEP operation has improved efficiency. With no flap loss or return to theater in the process mapping cohort, it has also demonstrated that this increased efficiency has been achieved without compromise to patient outcome. Moreover, 4/10 cases in cohort 1 had additional symmetrizing procedures (breast reduction/mastopexy) carried out concurrently compared with 0/10 cases in cohort 2. This demonstrates that additional procedures can be performed at the same time without increasing overall operative time significantly by using parallel operating techniques.

With increasing number of patients requiring breast reconstruction, there is a tightening of health care service purse strings. In this climate, as the clinicians performing these microsurgical procedures, it is mandatory that we look at the efficiency of ourselves carrying them out and understand any areas for improvement of quality of care. The principle of process mapping can be used in a similar fashion for all types of surgical procedures to maximize these benefits of reducing operative and therefore anesthetic time for the patients while also having time and cost benefits for the hospitals/trusts.^14^

Using the tools of process mapping, we have successfully broken down the DIEP operation in to a 100 steps. This has been used to create a template for the DIEP flap operation. Two cohort groups, one process-mapped and a control group, were timed for 10 unilateral DIEP flap reconstructions. The results from this have demonstrated that, by process mapping, efficiency of this operation has been improved. This has been achieved by carrying out the individual steps of the operation in a logical streamed manner with removal of redundant steps. This will hopefully dispel the impression of the DIEP flap operation being a long and complex operation to being a reproducibly straight-forward procedure when following the 100 steps. In addition to this, with the appropriate use of teamwork some of these steps can be carried out concurrently and therefore save even more time. The summative effect of this will be first to reduce the operative time of what was deemed to be a long operation for the patient to around 4 hours on average^7^ and thus reduce anesthetic time. Second, by saving time and with the efficient use of 2 or more teams additional procedures (eg, contralateral symmetrizing breast reductions) may be performed concurrently thus negating the requirement of a second operation and anesthetic for a patient. Third, reducing the operative time reproducibly to under 4 hours may enable multiple cases to be operated on in a day^6,7^ and thus maximize the use of theater time and as a result save costs while at the same time help with waiting lists. Further studies into process mapping the DIEP flap are likely to show its value in training by identifying the steps of most time variance thus elucidating areas of potential training focus and identifying any common areas of redundancy for trainees.

## CONCLUSIONS

Process mapping can be applied to DIEP flap surgery, as a means to evaluating operative efficiency and teaching. The 100 steps of the DIEP flap, as defined through evaluation of the operative approach of an experienced surgeon, are reproducible, are able to evaluate steps that have low or high variation between surgeons, and can be used to identify nonoperative steps between processes. The use of process mapping may thus be used to improve surgical technique, efficiency and in the future surgical training.
